# Topical Anesthetics Effectiveness in Management of Orthodontic Pain: A Systematic Review of Randomized Controlled Trials

**DOI:** 10.1002/cre2.70473

**Published:** 2026-10-06

**Authors:** Qasim Khalid, Abbas Naseem, Waqas Naseem, Adnan Jehangir, Umar Hussain, Maryam Altuhafy, Junad Khan

**Affiliations:** ^1^ Orofacial Pain and TMD, Eastman Institute for Oral Health University of Rochester Rochester New York USA; ^2^ Department of Orthodontics Niazi Medical and Dental College Sargodha Pakistan; ^3^ Department of Health Sciences University of Debrecen Debrecen Hungary; ^4^ BioMedical Sciences Department, College of Medicine King Faisal University Hofuf Saudi Arabia; ^5^ Saidu College of Dentistry Saidu Sharif Pakistan

**Keywords:** benzocaine, orthodontic pain, separator placement, systematic review, topical anesthetics

## Abstract

**Objectives:**

Orthodontic treatment can be associated with pain, particularly during separator placement, appliance insertion, or activation. Topical anesthetics may offer a simple, locally applied alternative to systemic analgesics, but their effectiveness remains uncertain. The objective of this review was to evaluate the efficacy of topical anesthetics, compared to other treatments or no treatment, in reducing orthodontic pain.

**Material and Methods:**

Eligible studies were randomized controlled trials (RCTs) comparing topical anesthetics (gels or patches) with placebo, no treatment, or other active pain‐control interventions in orthodontic patients. Five databases (PubMed, Scopus, Cochrane CENTRAL, Embase, Web of Science) were searched up to 14th March 2026, with no language or date restrictions. Data were extracted in duplicate, and the risk of bias was assessed using the ROB‐2 tool. Due to heterogeneity, only a qualitative synthesis was performed.

**Results:**

Nine RCTs (456 participants, mean age 14–35 years; 36.3% male) conducted in four countries were included. Benzocaine was the most investigated, followed by lidocaine, prilocaine/lidocaine, and menthol. Most studies examined separator‐induced pain. Eight of nine trials reported significant reductions in pain intensity with topical anesthetics at one or more time points, particularly within the first 24 to 72 h. Benzocaine consistently showed superior outcomes compared with placebo. Lidocaine and prilocaine/lidocaine provided rapid analgesia, especially in patients with high anxiety or low pain thresholds. Menthol patches also showed significant short‐term pain reduction in two studies. Risk of bias was moderate in most trials.

**Conclusion:**

Topical anesthetics can be effective in reducing short‐term orthodontic pain. Their use may reduce reliance on systemic analgesics and improve patient comfort during the initial stages of treatment. However, evidence quality remains limited, and further high‐quality RCTs with standardized protocols are needed.

## Highlights

- Systematic review of nine RCTs (*n* = 456) on topical anesthetics for orthodontic pain
- Benzocaine showed the most consistent pain reduction versus placebo
- Lidocaine and menthol gave rapid relief in anxious, low pain threshold patients
- Pain reduction was greatest within the first 24 to 72 h of application
- Evidence certainty was low to moderate; further high‐quality RCTs are needed

**Abbreviations**
CENTRAL: Cochrane Central Register of Controlled Trials
EMBASE: Excerpta Medica Database
GRADE: Grading of Recommendations Assessment, Development and Evaluation
LLLT: low‐level laser therapy
NSAIDs: nonsteroidal anti‐inflammatory drugs
PICOS: Population, Intervention, Comparison, Outcome, Study Design
PRISMA: Preferred Reporting Items for Systematic Reviews and Meta‐Analyses
RCTs: randomized controlled trials
ROB‐2: Risk of Bias 2 Tool
SD: standard deviation
TADs: temporary anchorage devices
TENS: transcutaneous electrical nerve stimulation
VAS: visual analog scale

## 1 Introduction

Pain and discomfort are common and often distressing experiences during orthodontic treatment, reported by as many as 90%–95% of patients following procedures such as separator placement, attachment bonding, or appliance activation (Tang et al. 2022; Sandhu et al. 2013). These symptoms arise from multiple factors, including periodontal ligament compression, release of inflammatory mediators, and mechanical trauma from brackets, wires, and separators (Rafeeq 2020; Long et al. 2016). Patients frequently describe the pain as soreness, pressure, or tightness, which typically peaks within the first 24 h and can persist for several days (Al‐Melh and Andersson 2017). Beyond physical discomfort, this pain may interfere with daily activities (Banerjee et al. 2018), reduce compliance, and overall acceptance of orthodontic therapy (Chow and Cioffi 2018), and about 30% patients even discontinued treatment prematurely (Nahidh and Alsaadi 2021).

Various strategies have been reported to alleviate orthodontic pain, ranging from invasive to non‐invasive approaches (Li et al. 2024). Systemic analgesics, primarily nonsteroidal anti‐inflammatory drugs (NSAIDs), remain the most common method (Cheng et al. 2020); however, concerns exist regarding their potential effects on tooth movement and adverse impacts on overall health (Walker and Buring 2001). Non‐pharmacological techniques, such as chewing wafers, vibratory stimulation, transcutaneous electrical nerve stimulation (TENS), and low‐level laser therapy (LLLT), have also been explored (Ren et al. 2022; Chawla et al. 2022).

Topical anesthetics are a non‐invasive, localized, and patient‐friendly method for reducing pain associated with orthodontic procedures (Eslamian et al. 2013). Preparations such as benzocaine gels, lidocaine/prilocaine eutectic mixtures, and medicated orthodontic waxes have been tested in various contexts (Al‐Melh and Andersson 2017). These agents act by blocking nociceptor transmission at the mucosal or gingival level, thereby reducing discomfort without systemic side effects. Previous studies have demonstrated the effectiveness of these procedures, including separator placement, archwire activation, and relief of mucosal irritation (Al‐Melh and Andersson 2017; Eslamian et al. 2013; Eslamian et al. 2016). However, findings across studies remain inconsistent regarding their comparative efficacy to placebo. Several clinical trials have investigated the use of topical anesthetics in orthodontics; however, the findings remain inconclusive due to variations in the agents used, study designs, and outcome measures. Since effective pain control is essential for patient comfort, compliance, and the overall success of treatment, it is crucial to consolidate the available evidence. This review compares topical anesthetics with other management or no treatments to clarify their role and effectiveness in managing orthodontic pain. The objective of this systematic review was to evaluate the efficacy of topical anesthetics, compared with placebo or other active interventions, in reducing orthodontic pain associated with separator placement, appliance insertion, or appliance activation.

## 2 Materials and Methods

### 2.1 Reporting Format

The current systematic review was conducted in accordance with the methodological principles outlined in the *Cochrane Handbook for Systematic Reviews of Interventions* (Higgins 2022). Reporting followed the *PRISMA 2020* statement (Page et al. 2021). To further ensure methodological transparency and prevent unnecessary duplication, the review protocol was prospectively registered in the International Prospective Register of Systematic Reviews (PROSPERO) under the identifier CRD420251130951. No meta‐analysis was performed due to heterogeneity in study designs, interventions, and outcome measures.

### 2.2 Focused Question

In orthodontic patients undergoing treatment, do topical anesthetics, compared with placebo, no treatment, or other interventions, reduce pain associated with orthodontic procedures?

### 2.3 Population, Intervention, Comparison, Outcome, Study Design (PICOS)

**P:** Orthodontic patients at the initiation of treatment or during active orthodontic therapy.

**I:** Topical anesthetics, including gels, patches, or waxes, applied before or after orthodontic procedures or appliance activation.

**C:** Placebo interventions (placebo gel, placebo patch, Vaseline, unmedicated orthodontic wax), no‐treatment controls, or other pain‐control methods such as low‐level laser therapy (LLLT), transcutaneous electrical nerve stimulation (TENS), ketoprofen, meloxicam, and diclofenac. Active comparators were included to assess the relative effectiveness of topical anesthetics compared with other pain‐management approaches.

**O:** Self‐reported pain intensity following orthodontic procedures.

**S:** Randomized controlled trials (RCTs).

### 2.4 Eligibility Criteria

The eligibility criteria for this systematic review were as follows: (1) Orthodontic patients, either at the initiation of treatment or during active treatment; (2) intervention group: use of topical anesthetics; (3) comparison group: comparisons were made with placebo and other interventions; (4) studies assessing self‐reported pain intensity; (5) RCTs. In terms of study design, case reports, case series, cohort studies, cross‐sectional studies, animal experiments, in‐vitro investigations, narrative reviews, and conference abstracts without sufficient data were excluded from consideration. Studies involving invasive orthodontic procedures, such as TAD placement, were excluded because these procedures commonly require infiltrative anesthesia and are associated with a different pain profile than routine orthodontic treatment. Both parallel‐group and split‐mouth randomized controlled trials were included.

### 2.5 Search Strategy and Study Selection Process

A comprehensive literature search was performed in five electronic databases (PubMed, Scopus, Cochrane CENTRAL, EMBASE, and Web of Science) using a predefined search strategy. The search spanned from database inception to August 14, 2025, and was updated on March 14, 2026, with no restrictions applied regarding publication date, language, study type, or publication status. Additionally, the reference lists of included studies and relevant systematic reviews were manually reviewed to identify further eligible studies. Additional search in Google Scholar and gray literature like Proquest was also done. Study selection was conducted independently by two authors (Q.K. and M.A.), and any discrepancies were resolved through discussion with a third author (J.K.).

### 2.6 Data Collection Process and Items

For each eligible study, data were extracted using a structured and pilot‐tested form to ensure consistency and completeness. The following key variables were collected: author and year of publication, country of origin, study design, sample size with gender distribution, mean or range of participant age, type of intervention(s) including specific topical anesthetics and comparators, and primary study findings related to pain reduction. Where available, details of control conditions (e.g., placebo gel, wax, or alternative interventions such as LASER or TENS) and outcome measures were also recorded. Pain intensity was typically assessed using validated scales such as the Visual Analog Scale (VAS), and both immediate and delayed effects were noted. Two reviewers (Q.K., M.A.) independently extracted the data, and any discrepancies were resolved through consensus or discussion with a third reviewer (J.K.). Because the included studies had shown considerable heterogeneity, a meta‐analysis could not be conducted.

### 2.7 Study Risk of Bias

All included studies were randomized controlled trials; therefore, the risk of bias was assessed using the revised Cochrane Risk of Bias tool for randomized trials (ROB‐2) (Sterne et al. 2019). The assessment was performed independently by two reviewers across five domains: bias arising from the randomization process, bias due to deviations from intended interventions, bias due to missing outcome data, bias in measurement of outcomes, and bias in selection of the reported result. Information regarding sequence generation, allocation concealment, blinding of participants and outcome assessors, completeness of follow‐up, and selective reporting was evaluated for each study. Each domain was judged as low risk, some concerns, or high risk of bias according to ROB‐2 guidance, and an overall risk of bias judgment was assigned for every included study. Any disagreements between reviewers were resolved through discussion with a third reviewer.

### 2.8 Synthesis Measures

Because of important differences among the included studies, a meta‐analysis was not carried out. Although some trials assessed similar comparisons, such as benzocaine versus placebo after separator placement using VAS scores, there were considerable variations in anesthetic concentration, formulation, mode of delivery, orthodontic procedures, application protocols, follow‐up periods, and pain assessment methods. In addition, several studies did not provide complete statistical information, such as standard deviations or change‐score data, required for calculation of standardized mean differences. Subgroup pooling based on intervention type and orthodontic procedure was explored; however, the small number of clinically comparable studies prevented meaningful quantitative synthesis. Therefore, the findings were summarized narratively following the Synthesis Without Meta‐analysis (SWiM) guidance (Campbell et al. 2020) to provide a transparent and structured presentation of the available evidence.

### 2.9 GRADE Analysis

The certainty of evidence for each outcome was assessed using the Grading of Recommendations Assessment, Development and Evaluation (GRADE) approach. Two reviewers independently assessed the evidence across the domains of risk of bias, inconsistency, indirectness, imprecision, and publication bias. Disagreements were resolved through discussion with a third reviewer. Downgrading decisions were made according to standard GRADE recommendations, and the reasons for each judgment were documented. The GRADEpro Guideline Development Tool (GRADEpro GDT) was used to prepare the Summary of Findings table and certainty ratings.

## 3 Results

### 3.1 Study Search

Presents the search strategies and results for each database, with the last search performed on 14th March 2026. The comprehensive search combined terms related to orthodontic interventions, pain, and topical anesthetics. This yielded 912 records from PubMed, 1672 from Scopus, 585 from Cochrane CENTRAL, 263 from Embase, and 27 from Web of Science. In Scopus, Cochrane CENTRAL, and Embase, searches were restricted to titles, abstracts, and keywords; in contrast, Web of Science searches included all fields.

### 3.2 Study Selection

A total of 3459 records were initially identified from electronic databases, with one additional record retrieved from gray literature. After removing 519 duplicates, 2941 records were screened based on title and abstract, of which 2925 records were excluded. The full texts of 16 articles were assessed for eligibility. Among these, 5 studies were excluded due to ineligible comparators, 1 for an ineligible population, and 1 was irrelevant, leaving 9 studies that were ultimately included in the review (Figure 1).

**Figure 1:**
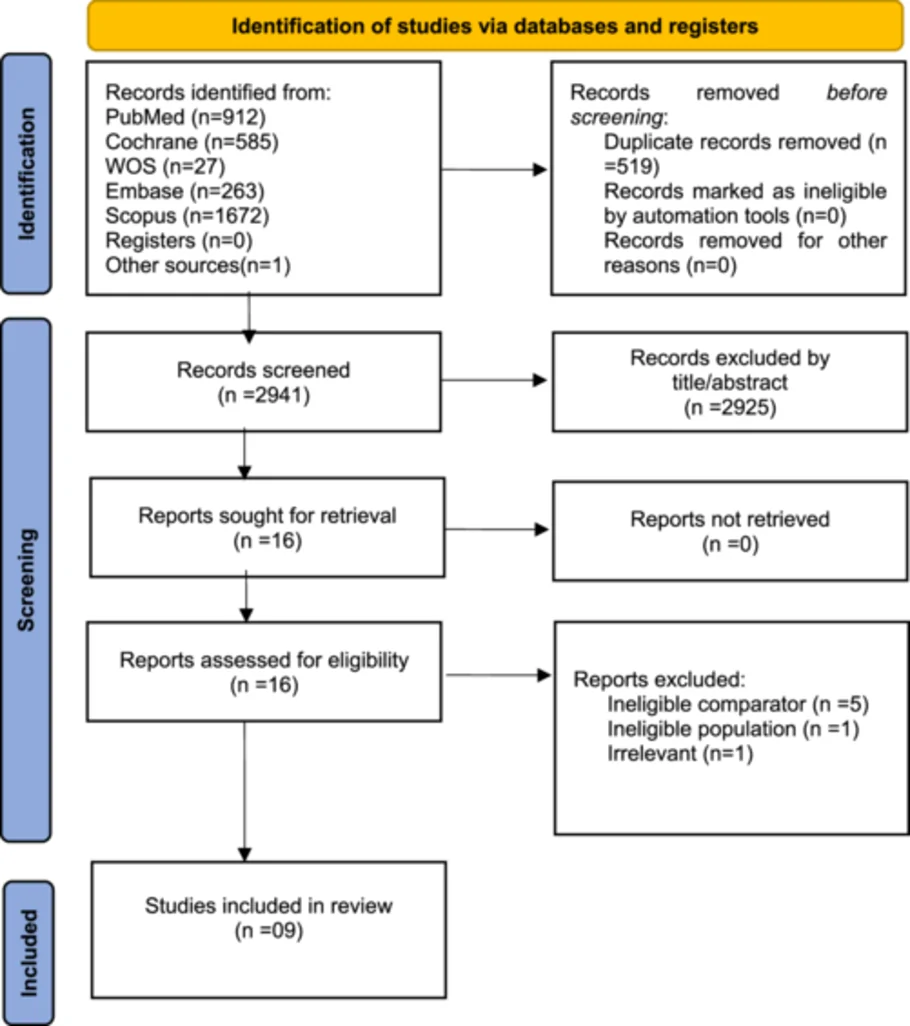
PRISMA flowchart of the study selection process.

### 3.3 General Characteristics

A total of 9 randomized controlled trials (RCTs) were identified, conducted across four countries: Iran, Pakistan, India, and the United States. Studies by Kluemper et al. (2002), Oza et al. (2020), and Paul (2023) had parallel groups. Collectively, these studies included 456 participants, with sample sizes ranging from 20 to 120. A study by Kluemper et al. (2002) did not specify any age group; however, given that the patients were treated for fixed orthodontic treatment, the minimum age considered was 12 years. Gender distribution was reported in six studies, males were comprised approximately 36.3% of the participants. Zakai et al. (2024), Eslamian (2016), Kluemper et al. (2002), and Paul (2023) did not report any gender distribution. The mean age across studies ranged from 14 to 35 years, with several trials including both adolescent and adult cohorts. Whereas the studies by Zakai et al. (2024), Eslamian et al. (2013) Eslamian (2016), Eslamian et al. (2016), and Soheilifar (2022a, 2022b) had a split‐mouth design. The experimental group included topical anesthetics in all the studies. Benzocaine represented the most extensively investigated intervention examined in seven studies (Eslamian et al. 2013; Eslamian et al. 2016; Eslamian 2016; Soheilifar 2022a; Oza et al. 2020; Kluemper et al. 2002; Paul 2023) at concentrations varying between 5% (Eslamian et al. 2016; Eslamian 2016) and 20% (Kluemper et al. 2002; Oza et al. 2020; Eslamian et al. 2013; Soheilifar 2022a). In contrast, Paul (2023) didn't mention the concentration of benzocaine that was used in the study. Additional topical agents included lidocaine (Zakai et al. 2024) and menthol (Soheilifar 2022a, 2022b). In addition to topical anesthetics, the study by Eslamian et al. (2016) had ketoprofen (topical analgesic), Oza et al. (2020) had LASER and TENS and Paul (2023) had meloxicam and diclofenac in the experimental group. The control group in studies by Zakai et al. (2024), Eslamian (2016), Eslamian et al. (2016), Eslamian et al. (2013), Paul (2023), Soheilifar (2022a, 2022b) was placebo gel or patch. Unmedicated wax was used by Kluemper et al. (2002), and the control group was only observed by Oza et al. (2020). Only two studies by Soheilifar (2022b, 2022a) mentioned the duration of the study, which was 2017–2019 and May 2018–Jan. 2019, respectively. None of the study was funded (Table 1).

**Table 1:** General characteristics of the included studies.

Author	Country	Study design	Number of participants	Mean ± SD age in years (range)	Gender M/F number	Study group	Control group	Study duration
Eslamian et al. (2013)	Iran	RCT	30	23 ± 3.75	18/12	Benzocaine 20%	Placebo patches	N/A
Zakai et al. (2024)	Pakistan	RCT	30	17.53 ± 2.03	N/A	2% Lidocaine	Placebo gel	N/A
Soheilifar (2022a)	Iran	RCT	64	14–25	25/39	Benzocaine menthol	Placebo	2017–2019
Eslamian et al. (2016)	Iran	RCT (cross over)	20	15–25	7/13	Benzocaine 5% ketoprofen	Placebo	N/A
Oza et al (2020)	India	RCT	120	18.04 ± 3.4	47/73	20% benzocaine LASER TENS	Placebo	N/A
Eslamian (2016)	Iran	RCT	30	15–25	N/R	Benzocaine 5%	Placebo	N/A
Kluemper et al. (2002)	USA	RCT	70	Adult grp > 18 Child grp < 18	N/R	Benzocaine 20%	Placebo gel	N/A
Paul (2023)	India	RCT	60	N/R	N/R	Benzocaine meloxicam diclofenac	Placebo	N/A
Soheilifar (2022b)	Iran	RCT	32	14–25	15/17	Menthol	Placebo	May 2018–Jan. 2019

Abbreviations: C, control group; E, experimental group; F, female; M, male; NR, not reported; TENS, transcutaneous electrical nerve stimulation; yrs, years.

### 3.4 Interventions in the Included Studies

As shown in Table 2a, most trials investigated the effects of separator‐induced pain. Six (Eslamian et al. 2013; Zakai et al. 2024, Soheilifar 2022a, Oza et al. 2020, Paul 2023) studies used elastomeric separators, whereas one trial (Soheilifar 2022b) assessed brass wire separators. Other orthodontic procedures included space closure with loops (Eslamian et al. 2016), archwire activation during alignment (Eslamian 2016), and bracket bonding (Kluemper et al. 2002). In terms of application mode, seven studies (Eslamian et al. 2013; Eslamian et al. 2016; Eslamian 2016; Soheilifar 2022a, 2022b; Kluemper et al. 2002; Paul 2023) used patient‐applied anesthetics, while three studies (Zakai et al. 2024; Oza et al. 2020) involved operator application. Benzocaine was the most frequently evaluated agent, reported in seven trials (70%), in either 5% or 20% formulations. Application sites varied but most commonly involved the buccal attached gingiva or interdental embrasures. Timing of application was immediately after (Eslamian et al. 2013; Soheilifar 2022a; Paul 2023, Soheilifar 2022b), few minutes after (Oza et al. 2020) or before (Zakai et al. 2024) separator placement, or after appliance activation/bonding (Eslamian 2016; Kluemper et al. 2002; Eslamian et al. 2016). Frequency of use ranged from a single one‐time application (Zakai et al. 2024; Oza et al. 2020) to repeated use every 6 h for 3 days (Eslamian et al. 2013; Soheilifar 2022a, 2022b), with others applying twice daily for 3 days (Eslamian 2016; Eslamian et al. 2016), or replacement at 48–72 h (Paul 2023). Lidocaine‐based formulations (lidocaine 2% or lidocaine/prilocaine) were used in two operator‐applied studies (Zakai et al. 2024), both applied 2 min before separator placement as a single dose. Menthol patches were tested in two studies (Soheilifar 2022a, 2022b), applied by patients to mandibular molars either immediately after separator placement or at 6‐h intervals for 3 days.

**Table 2a:** Details of interventions in the included studies.

Author	Diagnosis	Procedure	Type/Dosage	Applier	Application site	Timing	Frequency
Eslamian et al. (2013)	Patients with malocclusion scheduled for orthodontic treatment	Elastomeric separators	Benzocaine 20%	Patient	Buccal attached gingiva and embrasure of either the right or left first molar	Immediately after treatment	Repeat every 6 h for 3 days
Zakai et al. (2024)	Patients with malocclusion scheduled for orthodontic treatment	Elastomeric separators	Lidocaine 2%	Operator	Around the gingival margins, and into the crevices of the first and second premolar teeth	2 min before treatment	One‐time application
Soheilifar et al. (2022a)	Patients with malocclusion scheduled for orthodontic treatment	Elastomeric separators	Benzocaine/NR	Patient	Half patch on the tooth and half over the attached gingiva of the mandibular permanent first molar	Immediately after treatment	Repeat every 6 h for 3 days
	Patients with malocclusion scheduled for orthodontic treatment	Elastomeric separators	Menthol/NR	Patient	Half patch on the tooth and half over the attached gingiva of the mandibular permanent first molar	Immediately after treatment	Repeat every 6 h for 3 days
Eslamian et al. (2016)	Orthodontic patients in the space closure phase	Space closure with loops	Benzocaine 5%	Patients	Gingiva (buccally and palatally) and the coronal aspect of the teeth	After 2 mm activation of loops	Twice a day for 3 days
Oza et al. (2020)	Patients with malocclusion scheduled for orthodontic treatment	Elastomeric separators	Benzocaine 20%	Operator	Buccal attached gingiva and the embrasure of the first molars of each quadrant covering an area of about 1.5 cm in diameter	After placing the separators for 4 min	One‐time application
Eslamian (2016)	Leveling and alignment phase of treatment.	Archwire activation during treatment	Benzocaine 5%	Patient	The gingival margin of teeth in a way that it covers one‐third of the gingival part of the teeth and 2–3 mm of the gingiva	After treatment	Twice a day for 3 days
Kluemper et al. (2002)	Orthodontic treatment started with brackets	Bonding of a fixed orthodontic appliance without an arch wire	Benzocaine 20%	Patient	Amount necessary to cover the bracket	24 h after bonding on a particular bracket that causes irritation	No more than four times a day
Paul et al. (2023)	Patients with malocclusion scheduled for orthodontic treatment	Elastomeric separators	Benzocaine	Patient	Buccal attached gingivae and the embrasure of both the first maxillary molars	After inserting separators	Replaced after 48 h and 72 h
Soheilifar et al. (2022b)	Moderate crowding in the mandibular arch with non‐extraction orthodontic treatment plan	Brass wire separators	Menthol	Patient	Attached gingiva of the mandibular permanent first molar on one side, half on tooth and a half over the gum	Immediately after placement of the separators	Every 6 h for 3 days

### 3.5 Comparators in the Included Studies

The comparator interventions across the included studies consisted of placebo patches, placebo gels, unmedicated wax, or no active treatment. Although the placebo type, application site, timing, and frequency varied between studies, all comparators were designed to mimic the intervention without providing an active therapeutic effect (Table 2b).

**Table 2b:** Details of comparison in the included studies.

Author	Diagnosis	Procedure	Type/dosage	Applier	Application site	Timing	Frequency
Eslamian et al. (2013)	Patients with malocclusion scheduled for orthodontic treatment	Elastomeric separators	Placebo patches	Patient	Buccal attached gingiva and embrasure of either the right or left first molar	Immediately after treatment	Repeat every 6 h for 3 days
Zakai (2024)	Patients with malocclusion scheduled for orthodontic treatment	Elastomeric separators	Placebo gel (Vaseline based)	Operator	Around the gingival margins, and into the crevices of the first and second premolar teeth	2 min before treatment	One‐time application
Soheilifar (2022a)	Patients with malocclusion scheduled for orthodontic treatment	Elastomeric separators	Placebo patches*	Patient	Half patch on the tooth and half over the attached gingiva of the mandibular permanent first molar	Immediately after treatment	Repeat every 6 h for 3 days
Eslamian et al. (2016)	Orthodontic patients in the space closure phase	Space closure with loops	Placebo gel^a^	Patients	Gingiva (buccally and palatally) and the coronal aspect of the teeth	After 2 mm activation of loops	Twice a day for 3 days
Oza et al. (2020)	Patients with malocclusion scheduled for orthodontic treatment	Elastomeric separators	Placebo (no treatment)	Operator	Buccal attached gingiva and the embrasure of the first molars of each quadrant covering an area of about 1.5 cm in diameter	After placing the separators for 4 min	One‐time application
Eslamian (2016)	Leveling and alignment phase of treatment	Archwire activation during treatment	Placebo gel^b^	Patient	The gingival margin of teeth in a way that it covers one‐third of the gingival part of the teeth and 2–3 mm of the gingiva	After treatment	Twice a day for 3 days
Kluemper et al. (2002)	Orthodontic treatment started with brackets	Bonding of a fixed orthodontic appliance without an arch wire	Unmedicated wax	Patient	Amount necessary to cover the bracket	24 h after bonding on a particular bracket that causes irritation	No more than four times a day
Paul et al. (2023)	Patients with malocclusion scheduled for orthodontic treatment	Elastomeric separators	Placebo patches	Patient	Buccal attached gingivae and the embrasure of both the first maxillary molars	After inserting separators	Replaced after 48 h and 72 h
Soheilifar et al. (2022b)	Moderate crowding in the mandibular arch with non‐extraction orthodontic treatment plan	Brass wire separators	Placebo^c^^,^ *	Patient	Attached gingiva of the mandibular permanent first molar on one side, half on tooth and a half over the gum	Immediately after placement of the separators	Every 6 h for 3 days

* Dichloromethane, polyvinyl pyrrolidone, propylene glycol, ethanol, hydroxyl propylene methylcellulose, and aspartame.

^a^ Carbomer 934P, methylparaben, propylparaben, glycerin, sodium hydroxide, ethanol, and distilled water.

^b^ Carbomer 934P, methylparaben, propylparaben, glycerin, sodium hydroxide, and orange essence.

^c^ Dichloromethane, polyvinyl pyrrolidone, propyleneglycol, ethanol, hydroxyl propylene methyl cellulose, and aspartame.

### 3.6 Outcomes Measurements in the Included Studies

All nine studies assessed pain using the visual analog scale (VAS), with two also incorporating verbal or numerical rating scales. In eight of the studies, the use of topical agents resulted in statistically significant reductions in pain at one or more time points. Eslamian et al. (2013) found notable reductions at 2, 18, 24, 48, and 72 h. Zakai et al. (2024) observed significant reductions at 10 and 15 min, especially among anxious patients with low pain thresholds. Soheilifar (2022a) showed that benzocaine significantly lowered pain at 2 and 6 h, while menthol was effective at 2, 6, and 24 h. Eslamian et al. (2016) reported that benzocaine produced pain scores intermediate between ketoprofen and placebo, with the difference between benzocaine and placebo reaching significance only at 24 h and day 2. Eslamian (2016) found that benzocaine gel reduced pain significantly only at 2 h after archwire activation, with no significant difference from placebo at later time points. Soheilifar (2022b) reported that menthol patches significantly reduced pain after placement of brass wire separators at several time points up to 72 h. Oza et al. (2020) demonstrated immediate pain relief (*p* < 0.001) only within first 24 h. Kluemper et al. (2002) found that benzocaine wax significantly reduced discomfort from 24 h onwards, with effects sustained up to 77 h. Similarly, Paul (2023) showed significant reductions with benzocaine mucoadhesive patches at 6, 48, and 72 h (Table 2b, 3).

**Table 3:** Details of the outcomes reported in the included studies.

Author	Pain scale	Assessor	Pain evaluation	Pain scale	Statistical analysis		Outcome	Follow‐up	Postop medication	Adverse effects
					Period	*p*‐value				
Eslamian et al. (2013)	VAS	Patient	Immediately after separator placement and after 2, 6, 12,18, 24, 48, and 72 h.	0–10 cm	0 h	0.163	Significant reduction in pain levels for most time intervals	N/R	N/R	N/R
					2 h	0.007				
					6 h	0.087				
					12 h	0.059				
					18 h	0.019				
					24 h	0.019				
					48 h	0.003				
					72 h	0.002				
Zakai et al. (2024)	VAS	Patient	Every 5 min for 15 min	0–100 mm	0 min	0.079	Effective in reducing pain, especially in pts with low pain thresholds and anxiety	Same day	N/R	N/R
					5 min	0.860				
					10 min	0.001				
					15 min	0.002				
Soheilifar (2022a)	VAS	Patient	2 h	0–10 cm	2 h	0.0005	Patients had significantly lower pain with benzocaine	At 72 h	N/A	N/A
			6 h		6 h	0.0004				
			12 h		24 h	0.069				
			24 h							
			36 h							
			48 h							
			72 h							
	VAS	Patient	2 h	0–10 cm	2 h	0.0084	Patients had significantly lower pain with menthol	At 72 h	N/R	N/R
			6 h		6 h	0.007				
			12 h		24 h	0.049				
			24 h							
			36 h							
			48 h							
			72 h							
Eslamian et al. (2016)	VAS	Patient	2 h	0–4	2 h	> 0.05	Benzocaine had an effect mid‐way between ketoprofen and the placebo. The highest pain scores were recorded 2 h following the force application, and decreased to the lowest scores after seven days.	After 2 h	N/R	N/R
			6 h		6 h	> 0.05		6 h		
			24 h		24 h	0.01		24 h		
			Day 2		Day 2	0.04		Day 2		
			Day 3		Day 3	> 0.05		Day 3		
			Day 7		Day 7	> 0.05		Day 7		
Oza et al. (2020)	VAS	Patient	5 min	0–10 cm	Immediately	< 0.001	The topical anesthetic gel was found to be more effective in reducing pain immediately after placing the elastomeric separators as compared to the LASER, TENS, and control groups.	5 min	N/R	N/R
			24 h		24 h	0.06		24 h		
			48 h		48 h	0.08		48 h		
			72 h		72 h	0.98		72 h		
			96 h		96 h	0.35		96 h		
Eslamian (2016)	VAS	Patient	2 h	0–6	2 h	< 0.05	Benzocaine gel caused a decrease in pain at 2 h. Peak pain rank was at 6 h for benzocaine gel, followed by a decline in pain perception from that point to day 7.	2 h	N/R	N/R
			6 h		6 h	< 0.55		6 h		
			24 h		24 h	< 0.32		24 h		
			Day 2		Day 2	< 0.37		Day 2		
			Day 3		Day 3	< 0.49		Day 3		
			Day 7		Day 7	< 0.30		Day 7		
Kluemper et al. (2002)	VAS	Patient	24 h	0–100	24 h	0.04	Benzocaine wax is effective in reducing the mucosal discomfort and pro‐vides immediate pain relief that increases with time	N/R	N/R	N/R
			25 h		25 h	0.07				
			41 h		41 h	0.0003				
			53 h		53 h	< 0.001				
			65 h		65 h	< 0.001				
			77 h		77 h	< 0.001				
Paul (2023)	VAS	Patient	0 h (30 min after patch insertion)	0–10 cm	0 h (30 min after patch insertion)	0.355	Benzocaine was most effective in reducing pain when compared with placebo, meloxicam, and diclofenac.	N/R	N/R	N/R
			6 h		6 h	< 0.001				
			12 h		12 h	0.78				
			18 h		18 h	0.44				
			24 h		24 h	0.301				
			48 h		48 h	< 0.001				
			72 h		72 h	< 0.001				
	NRS	Patient	0 h (30 min after patch insertion)	1–10 cm	0 h (30 min after patch insertion)	0.66				
			6 h		6 h	< 0.001				
			12 h		12 h	0.659				
			18 h		18 h	0.174				
			24 h		24 h	0.461				
			48 h		48 h	< 0.001				
			72 h		72 h	< 0.001				
Soheilifar (2022b)	VAS	Patient	Immediately after	0–10 cm	Immediately	0.065	Menthol significantly reduces pain	N/R	N/R	N/R
			2 h		2 h	0.001				
			6 h		6 h	0.001				
			12 h		12 h	0.04				
			24 h		24 h	0.76				
			36 h		36 h	0.572				
			48 h		48 h	0.161				
			72 h		72 h	1				

Abbreviations: NR, not reported; VAS, visual analog scale.

### 3.7 Descriptive Effect Estimates

Because a meta‐analysis could not be performed, mean VAS values are reported here descriptively wherever the original studies provided this data, to convey the magnitude of effect beyond *p*‐values alone. Eslamian et al. (2013) reported a reduction in mean VAS from 2.28 to 1.63 with benzocaine patches at the 24‐h peak. Soheilifar (2022a) reported a mean VAS of 1.47 with benzocaine and 1.86 with menthol, compared with 2.75 for placebo. Soheilifar (2022b) reported a mean VAS of 4.87 with menthol compared with 6.06 for placebo at the 2‐h peak using brass wire separators. Eslamian et al. (2016) reported mean VAS scores of 0.68 with ketoprofen gel, 0.89 with benzocaine gel, and 1.15 with placebo. Zakai et al. (2024) reported mean pain scores of 15.8 versus 27.6 at 10 min and 5.23 versus 11.07 at 15 min with 2% lidocaine gel compared with placebo. Kluemper et al. (2002), Oza et al. (2020), Paul (2023), and Eslamian (2016) reported only *p*‐values or ranked comparisons without corresponding means or standard deviations in the original publications, which precluded calculation of mean differences for these comparisons. This inconsistency in outcome reporting across the primary studies is a limitation of the underlying evidence base and is discussed further below.

### 3.8 Risk of Bias of Included Studies

The risk of bias assessment is summarized in Figures 2 and 3. Eight studies demonstrated a moderate risk of bias, with concerns identified in one or more domains, while two studies were rated as high risk of bias. Zakai et al. (2024) and Eslamian et al. (2016) were judged to be at high risk, primarily due to deficiencies in the randomization process and incomplete outcome data, respectively. In several trials, selective reporting bias was evident, mainly due to the absence of trial registration and inadequate reporting of randomization procedures, which limited transparency and reproducibility. The remaining studies were considered to have a moderate risk of bias, most commonly due to issues related to the randomization process, deviations from intended interventions, and selection of reported results.

**Figure 2:**
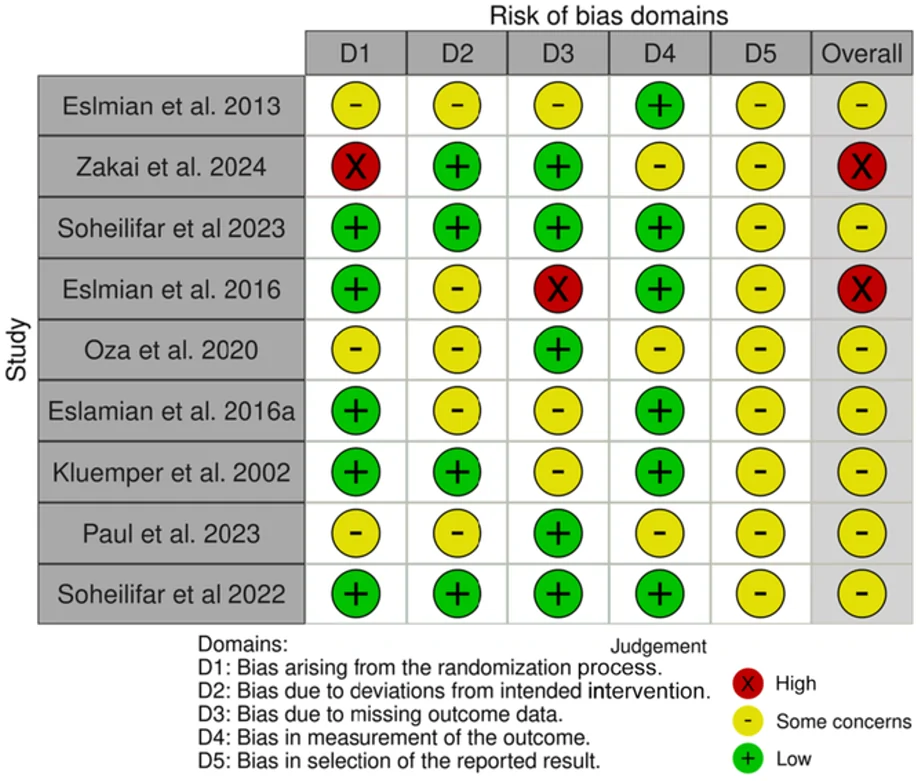
Risk of bias of the included studies using the ROB‐2 tool.

**Figure 3:**
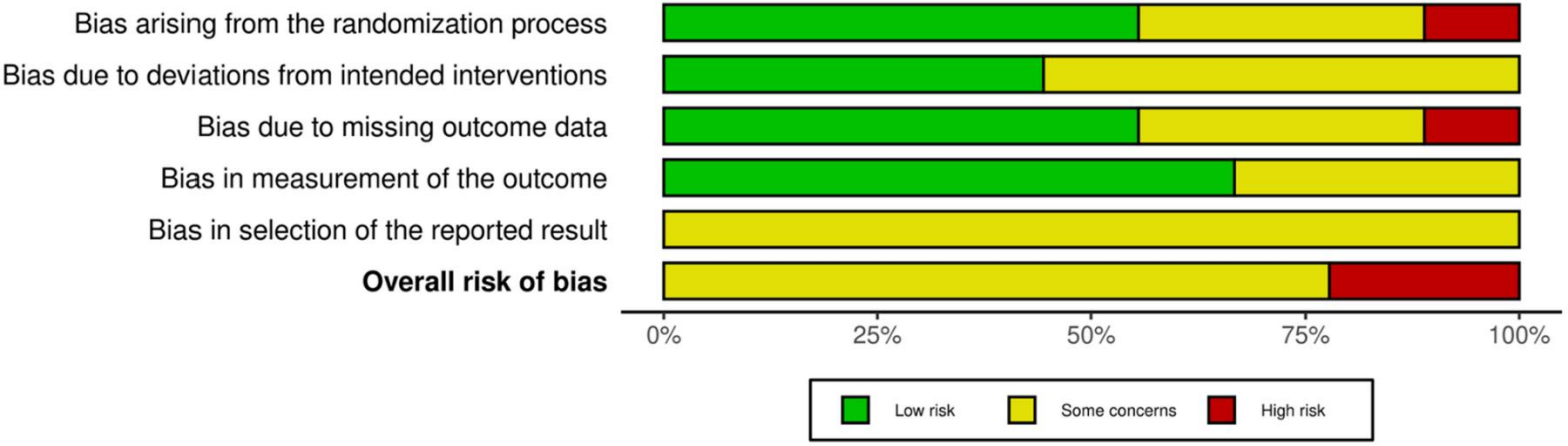
Summary plot of risk of bias.

### 3.9 GRADE Findings

The GRADE assessment of the nine included RCTs revealed moderate to very low certainty of evidence across all outcomes seen in Table 4. The highest certainty was assigned to benzocaine patch versus placebo and menthol patch versus placebo for pain reduction following orthodontic separator placement, supported by consistent findings across two to three studies with low to some concern for risk of bias. Evidence for the comparative efficacy of benzocaine versus menthol patches, NSAID patches (meloxicam and diclofenac), and benzocaine gel for archwire‐related pain was rated low, primarily due to heterogeneity in study designs, inconsistent results across dose levels and delivery methods, and small sample sizes. Very low certainty was assigned to ketoprofen gel and topical lidocaine gel, the former limited by a small crossover sample with significant dropouts and an archwire pain context not directly comparable to separator placement, and the latter constrained by a 15‐min follow‐up window that failed to capture the clinically relevant 12–24 h pain peak. No outcome reached high certainty, with single‐center conduct, limited sample sizes, and inconsistent blinding and allocation concealment procedures representing the most common downgrade factors across the body of evidence.

**Table 4:** GRADE analysis for the outcomes in the included studies.

Outcome	Number of studies	Key findings	Risk of bias	Certainty of evidence (GRADE)
Benzocaine patch versus placebo, VAS pain reduction (separators, 0–72 h)	3: Eslamian et al. 2013, Soheilifar (2022a), and Rahul Paul et al. (2023)	All three studies reported significantly lower VAS scores with benzocaine patches versus placebo (*p* < 0.001 to *p* = 0.019). Eslamian et al. found pain peaked at 24 h with benzocaine reducing mean VAS from 2.28 to 1.63. Soheilifar (2022a) reported mean benzocaine VAS of 1.47 versus placebo 2.75. l Paul et al. confirmed significant reduction at 6, 48, and 72 h. No adverse events across all three studies.	Some concern	⊕⊕⊕◯ Moderate^b^^,^ c
Menthol patch versus placebo, VAS pain reduction (separators, 0–72 h)	2: Soheilifar (2022a, 2022b)	Both RCTs showed significantly reduced pain with menthol patch versus placebo (*p* < 0.001). Soheilifar (2022a) reported mean menthol VAS of 1.86 versus placebo 2.75. Soheilifar (2022b) found maximum pain at 2 h (menthol 4.87 vs. placebo 6.06) using brass wire separators. Effect was significant in the first 12‐48 h and attenuated by 72 h. Pain resolved faster on the menthol side. No adverse events in either study.	Low	⊕⊕⊕◯ Moderate^a^^,^ b
Benzocaine versus menthol patch, comparative efficacy (separators)	2: Soheilifar et al. (2022a), Rahul Paul et al. (2023)	Benzocaine patches produced greater pain reduction than menthol in both studies, but the difference was not statistically significant (*p* = 0.115, Soheilifar 2022a). Rahul Paul et al. also ranked benzocaine highest when compared against meloxicam and diclofenac. Neither study was powered specifically for this head‐to‐head comparison.	Some concern	⊕⊕◯◯ Low^a^^,^ b ^,^ c
Benzocaine gel versus placebo, VAS pain reduction (archwire/appliance activation)	4: Eslamian et al. (2016), Eslamian (2016), Kluemper et al. 2002, and Oza et al. 2020	Mixed findings. Kluemper et al. (*n* = 70, benzocaine wax 20%) showed strong sustained reduction versus unmedicated wax (*p* ≤ 0.0003 at 4 of 6 time points). Oza et al. found benzocaine gel (20%, single dose) superior only immediately after placement, not beyond day 1. Eslamian et al. (2016) found mostly non‐significant differences from placebo, reaching significance only at 24 h and day 2 (*p* = 0.01 and *p* = 0.04). Eslamian (2016, 5% gel) showed a significant reduction only at 2 h (*p* < 0.05), with no significant difference thereafter. Dose and delivery method appear critical to efficacy.	Some concern	⊕⊕◯◯ Low^a^^,^ c
Meloxicam/diclofenac mucoadhesive patch versus placebo, VAS pain reduction (elastomeric separators)	1: Rahul Paul et al. (2023)	Rahul Paul et al. found both meloxicam and diclofenac patches significantly reduced pain versus placebo at 6, 48, and 72 h (*p* < 0.001). Diclofenac also significant at 12 h and 18 h. When all three drugs were compared, benzocaine ranked most effective, diclofenac second, and meloxicam third. Low VAS scores at 72 h across groups suggest natural pain resolution also contributes.	High concern	⊕⊕◯◯ Low^b^^,^ c
Ketoprofen gel versus benzocaine gel versus placebo, VAS pain reduction (archwire activation)	1: Eslamian et al. (2016	Eslamian et al. found ketoprofen gel (1.60 mg/mL) produced the lowest pain overall (mean 0.68), followed by benzocaine 5% (0.89), then placebo (1.15). Significant difference only between ketoprofen and placebo (*p* = 0.005); benzocaine versus placebo was non‐significant. Ketoprofen showed a consistent downward trend from 2 h to day 7; benzocaine effect was not sustained beyond 6 h.	Low	⊕◯◯◯ Very low^a^^,^ b ^,^ c
2% lidocaine gel versus placebo, VAS pain reduction (elastomeric separators, immediate 0–15 min)	1: Zakai et al. (2024)	Zakai et al. found 2% lidocaine gel significantly reduced pain versus Vaseline placebo at 10 min (15.8 vs. 27.6, *p* = 0.001) and 15 min (5.23 vs. 11.07, *p* = 0.002). No significant difference immediately or at 5 min. Effect significant only in the 14–18 age subgroup; non‐significant in 19–22 group. Critically, no data were collected beyond 15 min, the clinically relevant 12–24 h pain peak was not assessed.	Some concern	⊕◯◯◯ Very low^a^^,^ b ^,^ c
LLLT versus TENS versus benzocaine gel versus control, VAS pain reduction (elastomeric separators, days 1–4)	1: Oza et al. 2020	Oza et al. found LLLT produced the greatest pain reduction from day 1 to day 4 (*p* ≤ 0.001 on days 1–2). Benzocaine gel was superior immediately after placement only, with no benefit beyond day 1 (single‐dose protocol). TENS showed consistent but non‐significant improvement versus control. No gender difference detected across any group.	Some concern	⊕⊕◯◯ Low^a^^,^ c

Abbreviations: COI = conflict of interest, LLLT = low‐level laser therapy, PDL = periodontal ligament, TENS = transcutaneous electrical nerve stimulation.

^a^ Inconsistency.

^b^ Imprecision.

^c^ Risk of bias/study limitations. All included studies are RCTs (starting certainty = high). All were downgraded at least once.

## 4 Discussion

This systematic review synthesized evidence from randomized controlled trials evaluating topical anesthetics for orthodontic pain. Overall, the findings suggest that topical anesthetics may reduce orthodontic pain in the short term; however, the magnitude and duration of benefit varied according to the pharmacological agent, delivery vehicle, application protocol, orthodontic procedure, and timing of pain assessment (Eslamian et al. 2013; Eslamian et al. 2016; Campbell et al. 2020; Kluemper et al. 2002; Oza et al. 2020; Paul 2023; Zakai et al. 2024; Eslamian 2016; Soheilifar 2022a). Benzocaine was the most frequently investigated agent and showed the most consistent benefit, particularly when delivered as mucoadhesive patches, whereas gel‐based formulations demonstrated more variable and often transient effects (Eslamian et al. 2013; Eslamian et al. 2016; Kluemper et al. 2002; Oza et al. 2020; Paul 2023; Eslamian 2016; Soheilifar 2022a). Due to substantial heterogeneity among the included trials in terms of study design, intervention protocols, and outcome measures, a meta‐analysis was not performed (Campbell et al. 2020). Heterogeneity among studies was due to methodological factors, including risk of bias in randomization, outcome reporting, and blinding domain, as well as clinical variations like type, concentration, and method of application of topical anesthetics, the orthodontic procedures investigated (e.g., separator placement, space closure, archwire activation), and the timing of outcome assessments. Population‐related variables, including age, sex, and individual pain thresholds, may also have influenced treatment responses. These sources of heterogeneity limit comparability across trials and emphasize the need for standardized protocols and rigorous study methods in future research. Therefore, a narrative synthesis was performed, whereby the results of each randomized controlled trial were systematically appraised according to study design, timing of pain assessment, method of anesthetic administration, pharmacologic agent, and analgesic effect (Campbell et al. 2020).

The inclusion of different randomized controlled designs, including parallel‐group, split mouth, and crossover trials. Although split‐mouth and crossover designs can reduce inter‐individual variability, they may introduce methodological concerns related to within subject correlation, carry‐over effects, and non‐independence of observations. Therefore, findings from those studies should not be interpreted as directly equivalent to conventional parallel‐group randomized controlled trials (Eslamian et al. 2016; Eslamian 2016; Soheilifar 2022a).

Orthodontic procedures are often associated with pain, which can impair daily functioning and compromise patient compliance (Chow and Cioffi 2018; Krukemeyer et al. 2009). In this systematic review, six studies evaluated pain management during the placement of elastomeric separators (Al‐Melh and Andersson 2017; Eslamian et al. 2013; Eslamian et al. 2016; Oza et al. 2020; Zakai et al. 2024; Soheilifar 2022a). One study examined pain during the bonding of fixed appliances without archwire placement (Kluemper et al. 2002), while another assessed pain during space closure with loops (Eslamian 2016). Pain associated with archwire activation in the leveling and alignment phase was investigated in one study (Eslamian et al. 2016), and a further study evaluated the use of brass wire separators in patients with moderate mandibular crowding undergoing non‐extraction treatment (Soheilifar 2022b). Our findings showed that topical anesthetics may be helpful in many orthodontic procedures that are often associated with pain. Unlike systemic analgesics or device‐based modalities, such as low‐level laser therapy and TENS, (Ren et al. 2022) topical anesthetics offer a simple, non‐invasive, and well‐tolerated approach with minimal systemic side effects and invasiveness. However, their analgesic effect appears to be transient, mainly because these agents act only on superficial nerve endings at the mucosal surface and are rapidly diluted or removed by saliva, mastication, and swallowing (Schønemann et al. 1992). Consequently, the duration of anesthesia is limited to the period of direct mucosal absorption, which explains why most studies reported pain reduction confined to the first few hours after appliance activation.

Benzocaine, evaluated in seven of the included trials consistently demonstrated greater pain reduction compared with placebo (Eslamian et al. 2013; Eslamian et al. 2016; Kluemper et al. 2002; Oza et al. 2020; Paul 2023; Eslamian 2016; Soheilifar 2022a). Its rapid onset of action and localized delivery may account for this finding. Lidocaine‐based preparations also proved effective, particularly among anxious patients and those with low pain thresholds, indicating the potential value of customizing anesthetic selection to individual patient characteristics. Menthol patches, investigated in two studies (Soheilifar 2022a, 2022b), were associated with significant pain reduction; however, the limited number of trials restricts definitive conclusions about their clinical applicability. Study by Eslamian et al. (2016) concluded that effect of benzocaine was midway between placebo and ketoprofen, a topical analgesic. In this study, a significant difference between benzocaine and ketoprofen was found only at 24 h mark, favoring ketoprofen, while all other intervals indicated no significant difference (Eslamian et al. 2016). Whereas, Paul (2023) compared benzocaine with two topical analgesics (meloxicam and diclofenac) and reported that benzocaine was more effective than topical analgesics especially at 48th and 72nd hours. Oza et al. (2020) compared the effectiveness of 20% benzocaine, laser therapy, transcutaneous electrical nerve stimulation (TENS), and placebo in managing pain following the placement of elastomeric separators and found that benzocaine provided significantly greater pain relief immediately after separator placement compared with the other interventions However, this finding was based on a single study and should be interpreted cautiously (Oza et al. 2020).

The included studies used different comparator groups, including placebo controls, no‐treatment groups, topical NSAID preparations, TENS, and low‐level laser therapy. Placebo and no‐treatment comparisons were primarily intended to evaluate the analgesic efficacy of topical anesthetics alone, whereas comparisons with active interventions assessed their relative effectiveness against other pain‐management approaches. Because these comparators differ substantially in mechanism of action and clinical application, direct comparisons across studies should be interpreted cautiously.

The certainty of evidence varied from moderate to very low for the outcomes assessed in this review. Moderate‐quality evidence indicated that benzocaine and menthol patches could provide short‐term pain relief after separator placement. In contrast, the evidence related to lidocaine, ketoprofen, and several other topical agents was less conclusive because of limited sample sizes, variability in study findings, and methodological concerns within the included trials. Although many studies reported favorable outcomes with topical anesthetics, the overall strength of evidence remains limited.

Clinically, topical anesthetics may serve as a supportive measure for managing short‐term orthodontic pain, especially during separator placement and the initial stages of appliance activation. Their local mode of action, simple application, and reduced systemic exposure may be advantageous for patients who prefer to avoid systemic analgesics or have contraindications to their use. Nevertheless, the pain‐relieving effect appears to be short‐lived, and current evidence is insufficient to draw firm conclusions regarding long‐term efficacy and safety.

This systematic review has several strengths. To our knowledge, this is the first study to evaluate this topic in orthodontics. The review was conducted using rigorous methods based on the Cochrane Handbook for Systematic Reviews of Interventions and reported in accordance with PRISMA guidelines. A systematic search was conducted across all major electronic databases to collect all available evidence. Independent risk of bias assessments were performed in duplicate, the review was prospectively registered, and inclusion was limited to randomized controlled trials (RCTs), which helped reduce the risk of confounding that is common in observational studies.

However, several limitations should be considered. Many of the included trials showed a moderate to high risk of bias, often due to issues with randomization, blinding, and selective outcome reporting. There was also wide variation in the types of interventions studied, the outcomes measured, and the length of follow‐up, which prevented pooled quantitative analysis. Furthermore, fewer than half of the included trials reported mean pain scores or standard deviations alongside *p*‐values, so descriptive effect estimates could be presented for only some comparisons; this inconsistency in the primary literature limits judgments about clinical, as opposed to statistical, significance. Adverse effects were rarely reported, making it difficult to draw firm conclusions about the safety of topical anesthetics in orthodontic patients. In addition, most studies had relatively small sample sizes, which limits the extent to which the results can be applied to clinical practice. In addition, the inclusion of split‐mouth and crossover trials may have introduced bias related to carry‐over effects and within‐subject correlation, potentially affecting the precision of treatment‐effect estimates (Eslamian et al. 2016; Eslamian 2016; Soheilifar 2022a).

Future research should prioritize high‐quality randomized controlled trials using standardized treatment protocols, uniform pain assessment tools, and extended follow‐up durations. Additional studies comparing various topical anesthetic agents, formulations, and concentrations are also needed. Evaluating potential adverse effects alongside patient‐reported outcomes would provide more comprehensive evidence and support more reliable clinical decision‐making for orthodontic pain management.

## 5 Conclusion

Within the limitations of the available evidence, topical anesthetics, particularly benzocaine formulations, may provide short‐term reduction in orthodontic pain, especially following separator placement. However, the certainty of evidence remains low to moderate, and these findings should be interpreted with caution. Further well‐designed randomized controlled trials with standardized protocols, adequate blinding, and longer follow‐up are required.

## Author Contributions

**Qasim Khalid:** conceptualization (equal), data curation (equal), risk of bias (equal), investigation (equal), writing – review and editing (equal). **Abbas Naseem:** conceptualization (equal), data extraction (equal), writing – review and editing (equal). **Waqas Naseem:** data curation (equal), risk of bias (equal), investigation (equal), writing – review and editing (equal). **Adnan Jehangir:** conceptualization (equal), investigation (equal), methodology (equal), writing – review and editing (equal). **Umar Hussain:** conceptualization (equal), investigation (equal), methodology (equal), project administration (equal), writing – original draft (equal), software (equal), formal analysis (equal). **Maryam Altuhafy:** conceptualization (equal), investigation (equal), methodology (equal), writing – review and editing (equal). **Junad Khan:** data curation (equal), risk of bias (equal), GRADE analyses, supervision, writing – review and editing (equal).

## Funding

The authors have nothing to report.

## Consent

The authors have nothing to report.

## Conflicts of Interest

The authors declare no conflicts of interest.

## Supporting information


Supporting File

## Data Availability

No new data were generated or analyzed in this study. All data extracted for this systematic review are available within the published articles cited in the reference list.
