# Supplementary material for: Topical Anesthetics Effectiveness in Management of Orthodontic Pain: A Systematic Review of Randomized Controlled Trials
Source: Clin Exp Dent Res. 2026 Oct 6;12(5):e70473. doi: 10.1002/cre2.70473 (PMC13640979; doi:10.1002/cre2.70473)
Supplement: Supplementary file 1 [file CRE2-12-e70473-s001.docx]

# PRISMA 2020 Checklist

*Manuscript:* ***Topical Anesthetics Effectiveness in Management of Orthodontic Pain: A Systematic Review of Randomized Controlled Trials***

From: Page MJ, McKenzie JE, Bossuyt PM, et al. The PRISMA 2020 statement: an updated guideline for reporting systematic reviews. BMJ 2021;372:n71.

| **Section/Topic** | **#** | **Checklist item** | **Location in manuscript** |
| --- | --- | --- | --- |
| **TITLE** | | | |
| Title | 1 | Identify the report as a systematic review. | Title page |
| **ABSTRACT** | | | |
| Abstract | 2 | See the PRISMA 2020 for Abstracts checklist. | Abstract |
| **INTRODUCTION** | | | |
| Rationale | 3 | Describe the rationale for the review in the context of existing knowledge. | Introduction, paragraphs 1-2 |
| Objectives | 4 | Provide an explicit statement of the objective(s) or question(s) the review addresses. | Materials and Methods – Focused Question |
| **METHODS** | | | |
| Eligibility criteria | 5 | Specify the inclusion and exclusion criteria for the review and how studies were grouped for the syntheses. | Materials and Methods – Eligibility criteria; PICOS |
| Information sources | 6 | Specify all databases, registers, websites, organisations, reference lists and other sources searched or consulted, and the date each was last searched. | Materials and Methods – Search strategy and study selection process |
| Search strategy | 7 | Present the full search strategies for all databases, registers and websites, including filters and limits used. | Materials and Methods – Search strategy and study selection process |
| Selection process | 8 | Specify the methods used to decide whether a study met the inclusion criteria, including number of reviewers, whether independent, and use of automation tools. | Materials and Methods – Search strategy and study selection process |
| Data collection process | 9 | Specify the methods used to collect data from reports, including number of reviewers, whether independent, and use of automation tools. | Materials and Methods – Data collection process and items |
| Data items | 10a | List and define all outcomes for which data were sought. | Materials and Methods – PICOS; Data collection process and items |
| Data items | 10b | List and define all other variables for which data were sought. | Materials and Methods – Data collection process and items |
| Study risk of bias assessment | 11 | Specify the methods used to assess risk of bias in the included studies, including tool(s) used and number/independence of reviewers. | Materials and Methods – Study risk of bias |
| Effect measures | 12 | Specify for each outcome the effect measure(s) used in the synthesis or presentation of results. | Materials and Methods – Synthesis measures |
| Synthesis methods | 13a | Describe the processes used to decide which studies were eligible for each synthesis. | Materials and Methods – Synthesis measures |
| Synthesis methods | 13b | Describe methods required to prepare the data for synthesis. | Materials and Methods – Synthesis measures |
| Synthesis methods | 13c | Describe methods used to tabulate or visually display results. | Results – Outcomes measurements; Table 3 |
| Synthesis methods | 13d | Describe methods used to synthesize results and the rationale; meta-analysis model(s) and software if used. | Materials and Methods – Synthesis measures (narrative synthesis; meta-analysis not performed due to heterogeneity) |
| Synthesis methods | 13e | Describe methods used to explore possible causes of heterogeneity. | Discussion (heterogeneity across trials); not applicable for meta-regression/subgroup analysis, as no meta-analysis was performed |
| Synthesis methods | 13f | Describe any sensitivity analyses conducted. | Not applicable – no meta-analysis was performed |
| Reporting bias assessment | 14 | Describe methods used to assess risk of bias due to missing results (reporting biases). | Materials and Methods – GRADE Analysis |
| Certainty assessment | 15 | Describe methods used to assess certainty (confidence) in the body of evidence for an outcome. | Materials and Methods – GRADE Analysis |
| **RESULTS** | | | |
| Study selection | 16a | Describe results of the search and selection process, from records identified to studies included, ideally with a flow diagram. | Results – Study search; Study selection; Fig 1 (PRISMA flow diagram) |
| Study selection | 16b | Cite studies that might appear to meet inclusion criteria but were excluded, and explain why. | Results – Study selection (full-text exclusions and reasons) |
| Study characteristics | 17 | Cite each included study and present its characteristics. | Results – General characteristics; Table 1 |
| Risk of bias in studies | 18 | Present assessments of risk of bias for each included study. | Results – Risk of bias of included studies; Fig 2, Fig 3 |
| Results of individual studies | 19 | For all outcomes, present summary statistics and effect estimates with precision for each study. | Results – Outcomes measurements in the included studies; Table 3 |
| Results of syntheses | 20a | Briefly summarise the characteristics and risk of bias among studies contributing to each synthesis. | Discussion, paragraph 1 |
| Results of syntheses | 20b | Present results of all statistical syntheses conducted. | Results – Outcomes measurements in the included studies (narrative synthesis; meta-analysis not performed) |
| Results of syntheses | 20c | Present results of investigations of possible causes of heterogeneity. | Discussion (sources of heterogeneity) |
| Results of syntheses | 20d | Present results of any sensitivity analyses. | Not applicable – no meta-analysis was performed |
| Reporting biases | 21 | Present assessments of risk of bias due to missing results for each synthesis. | Results – Risk of bias of included studies (selective reporting) |
| Certainty of evidence | 22 | Present assessments of certainty in the body of evidence for each outcome. | Results – GRADE findings; Table 4 |
| **DISCUSSION** | | | |
| Discussion | 23a | Provide a general interpretation of results in the context of other evidence. | Discussion, paragraphs 1-3 |
| Discussion | 23b | Discuss limitations of the evidence included in the review. | Discussion; GRADE findings |
| Discussion | 23c | Discuss limitations of the review processes used. | Discussion, final paragraphs |
| Discussion | 23d | Discuss implications of the results for practice, policy and future research. | Discussion, final paragraph; Conclusion |
| **OTHER INFORMATION** | | | |
| Registration and protocol | 24a | Provide registration information, including register name and registration number. | Materials and Methods – Reporting format (PROSPERO CRD420251130951) |
| Registration and protocol | 24b | Indicate where the review protocol can be accessed. | Materials and Methods – Reporting format |
| Registration and protocol | 24c | Describe and explain any amendments to information provided at registration or in the protocol. | Not applicable – no amendments were made to the registered protocol |
| Support | 25 | Describe sources of financial or non-financial support, and role of funders/sponsors. | Declaration – Funding |
| Competing interests | 26 | Declare any competing interests of review authors. | Declaration – Conflict of interest statement |
| Availability of data, code and other materials | 27 | Report which of the following are publicly available and where: data collection forms, extracted data, analytic code, other materials. | Declaration – Data Availability Statement |
